# Cerebrospinal fluid lactate is associated with multiple sclerosis disease progression

**DOI:** 10.1186/s12974-016-0502-1

**Published:** 2016-02-10

**Authors:** Maria Albanese, Sara Zagaglia, Doriana Landi, Laura Boffa, Carolina G. Nicoletti, Maria Grazia Marciani, Georgia Mandolesi, Girolama A. Marfia, Fabio Buttari, Francesco Mori, Diego Centonze

**Affiliations:** Multiple Sclerosis Clinical and Research Unit, Department of Systems Medicine, Tor Vergata University, Via Montpellier 1, 00133 Rome, Italy; IRCCS Istituto Neurologico Mediterraneo (INM) Neuromed, 86077 Pozzilli, IS Italy; Clinica di Neurologia, Università Politecnica delle Marche, 60100 Ancona, Italy; IRCCS Fondazione Santa Lucia, 00143 Rome, Italy

**Keywords:** CSF, EDSS, Inflammation, Mitochondrial damage, MS, Neurofilaments, Neurodegeneration, Tau protein

## Abstract

**Background:**

Altered cerebrospinal fluid (CSF) levels of lactate have been described in neurodegenerative diseases and related to mitochondrial dysfunction and neuronal degeneration. We investigated the relationship between CSF lactate levels, disease severity, and biomarkers associated with neuroaxonal damage in patients with multiple sclerosis (MS).

**Methods:**

One-hundred eighteen subjects with relapsing-remitting multiple sclerosis (RRMS) were included, along with one-hundred fifty seven matched controls. CSF levels of lactate, tau protein, and neurofilament light were detected at the time of diagnosis. Patients were followed-up for a mean of 5 years. Progression index (PI), multiple sclerosis severity scale (MSSS), and Bayesian risk estimate for multiple sclerosis (BREMS) were assessed as clinical measures of disease severity and progression. Differences between groups and correlation between CSF lactate, disease severity and CSF biomarkers of neuronal damage were explored.

**Results:**

CSF lactate was higher in RRMS patients compared to controls. A negative correlation was found between lactate levels and disease duration. Patients with higher CSF lactate concentration had significantly higher PI, MSSS, and BREMS scores at long-term follow-up. Furthermore, CSF lactate correlated positively and significantly with CSF levels of both tau protein and neurofilament light protein.

**Conclusions:**

Measurement of CSF lactate may be helpful, in conjunction with other biomarkers of tissue damage, as an early predictor of disease severity in RRMS patients. A better understanding of the alterations of mitochondrial metabolic pathways associated to RRMS severity may pave the way to new therapeutic targets to contrast axonal damage and disease severity.

## Background

Multiple sclerosis (MS) is a chronic autoimmune disorder of the central nervous system (CNS), presenting with unpredictable clinical relapses and remissions and by disability progression over time [1]. Neuropathologically, MS is characterized by an inflammatory reaction in close relationship with diffuse neurodegenerative processes [2]. Experimental evidence suggests that astroglial activation and axonal damage are both present in the early stages of the disease, leading to neuronal injury, inflammatory demyelination, and neurodegeneration [3, 4].

In the last years, mitochondrial dysfunction and subsequent energy penalty have been hypothesized to drive axonal degeneration and disease progression [5–7]. In this context, neuronal lactate secretion in the cerebrospinal fluid (CSF) of multiple sclerosis (MS) subjects has been investigated with controversial results reporting either increased, decreased, or unchanged levels [8–13]. Major limitations of the published MS studies are the small sample sizes and high variability of patient cohorts.

Recent data indicate that other biomarkers associated with neuroaxonal damage, such as tau protein (t-tau), play an important role in modulating mitochondrial function and dynamics [14]. To date, the impact of these neuronal markers on mitochondrial function and energy metabolism has never been explored in MS.

The aim of our study was to investigate the following: (1) the level of CSF lactate in patients with relapsing-remitting multiple sclerosis (RRMS) compared to healthy subjects; (2) the potential value of CSF lactate to predict disease severity and progression; (3) the relationship between CSF lactate levels and both t-tau and neurofilament light (NFL) protein.

## Methods

The study was approved by the ethics committee of the Tor Vergata Hospital in Rome, and informed consent was obtained from all patients.

### Study design

We collected CSF from 118 consecutive patients admitted to the Neurology Clinic of Tor Vergata Hospital of Rome and diagnosed as RRMS according to validated criteria [15]. Subjects enrolled in the study were treated with immunomodulatory or immunosuppressive therapies always after CSF collection. Patients receiving steroids in the 30 days before lumbar puncture (LP) were not included.

One hundred fifty-seven age- and gender-matched individuals, who underwent neurological investigation, brain magnetic resonance imaging (MRI), and LP for diagnostic purposes, were enrolled as control subjects. They resulted negative for inflammatory or degenerative disorders of the central and peripheral nervous system, systemic diseases, and of abnormal cell count and/or other abnormalities in the CSF. Subjects under treatment with drugs interfering with central nervous system (CNS) were also excluded.

At the time of confirmed diagnosis, all MS patients started disease-modifying therapy. Second-line treatment was also considered for patients who experienced at least two relapses during 1 year of therapy with other approved immunomodulatory agents.

After their admittance, all patients underwent CSF and blood tests, complete neurological evaluation, and brain (and in selected cases also spinal) MRI scan. Clinical status was assessed during outpatient scheduled visits every 3 months. Demographic and clinical data were collected from medical records. MS disease onset was defined as the first episode of focal neurological dysfunction suggestive of MS. Disease duration was estimated as the number of years from onset to the most recent assessment of disability. Clinical activity was defined as the occurrence of any clinical relapse, during the follow-up period.

Relapses were defined as the occurrence of new or recurrent neurological symptoms not associated with fever or infection lasting for at least 24 h. The annualized relapse rate (ARR) was defined as the number of relapses per year. In addition, the number of relapses in the first 2 years of the disease course and the time until the first relapse were used as clinical indexes of inflammatory activity. Disability was determined by a trained neurologist using the expanded disability status scale (EDSS) (http://www.neurostatus.net/index.php?file=start), a ten-point scale measuring neurological disability by rating nine different neurological domains [16]. Sustained EDSS progression was defined as a one-point increase persisting for at least 6 months. The EDSS score, assessed every 6 months after diagnosis, was used in combination with disease duration to calculate two measures of disease severity: the progression index (PI) and the multiple sclerosis severity scale (MSSS). The PI was defined as the current EDSS score divided by disease duration expressed in years. The MSSS is an algorithm that relates EDSS scores to distribution of disability in patients with comparable disease duration [17]. MSSS has advantages over the PI such as being more stable over time and more accurate when comparing disease severity using single assessment data. We also considered the Bayesian risk estimate for multiple sclerosis (BREMS) score that was calculated using gender, age at onset, and clinical events during the first year of the disease to identify individual risk of secondary progression [18].

### CSF sampling and analyses

All CSF samples were obtained through LP performed in lateral decubitus. CSF samples were collected in polypropylene tubes using standard sterile techniques. Each CSF sample was divided in two aliquots: 2 ml of CSF sample were used for biochemistry analysis including total cell count and lactate levels; 3 ml of CSF sample were centrifuged at 1300 rpm for 10 min after withdrawal to remove cellular elements and immediately stored at −80 °C until used. Biochemistry assays were carried out using commercially available kits following the manufacturer’s instruction. NFL was detected in CSF samples using commercial ELISA kit (Uman Diagnostics NF-light® assay, Umea, Sweden). The levels of NFL in CSF were measured by fitting data to a four-parameter standard curve using GraphPad Prism Software Package (San Diego, CA, USA).CSF levels of t-tau were quantified with standard procedures, using commercially available ELISA test (FujireBio, Tokyo, Japan). The biomarker concentrations were calculated using a standard sigmoid curve equation (www.fdi.com).

### Statistical analysis

Data distribution was analyzed through Kolmogorov-Smirnov test. Between group comparisons of CSF lactate, NFL and t-tau levels were performed by means of Mann-Whitney test. Within the MS group, the relationships between the CSF levels of lactate, NFL, and t-tau with other clinical and radiological variables were evaluated using Spearman’s correlation analysis and partial correlation analysis to adjust the results for age and gender. *P* value of <0.05 was considered to be statistically significant.

## Results

The demographic features and clinical characteristics of RRMS patients are shown in Table 1. The median follow-up duration was 5 years. EDSS values ranged from 0 to 3.5.

**Table 1:** Demographic and clinical characteristics of enrolled subjects

Variable	Subject group	
	Control subjects (*n* = 157)	Multiple sclerosis (*n* = 118)
Gender (M/F)	40/117	38/80
Age (years)	40 ± 15.23	31.3 ± 9.14
Disease duration (years)	N/A	7 ± 5.86
EDSS	N/A	1.25 ± 0.74

Data are mean ± standard deviation

*M* male, *F* female, *EDSS* expanded disability status scale, *N/A* not applicable

### CSF lactate levels are higher in RRMS patients compared to controls

**Fig. 1:**
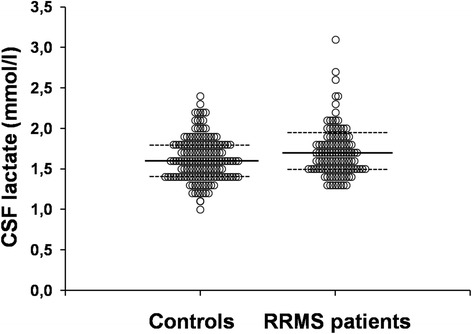
CSF lactate levels are higher in RRMS patients compared to controls. Dot plots of CSF lactate concentration data distribution for relapsing-remitting MS (*RRMS*, *n* = 118) and healthy control subjects (CTRL, *n* = 157). *Horizontal bars* represent group mean; *dotted lines* represent quartiles

CSF lactate was higher in RRMS patients compared to controls (*p* = 0.008) (Fig. 1). Lactate levels correlated with age at the time of LP both in the RRMS (*r*_s_ = 0.19; *p* = 0.001) and control (*r*_s_ = 0.30; *p* < 0.001) groups (not shown).

### CSF lactate levels correlate with future measures of disease severity

**Fig. 2:**
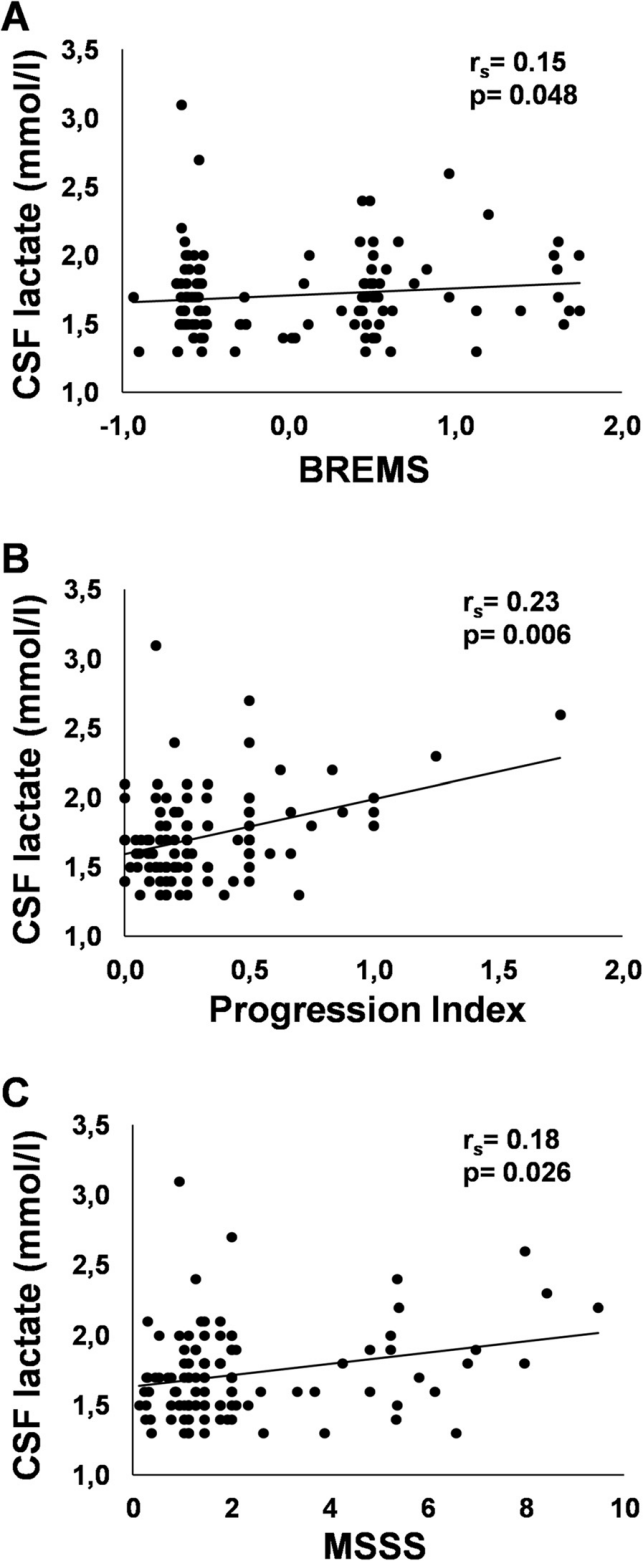
CSF lactate levels correlate with future measures of disease severity in RRMS patients. Lactate levels in CSF collected during diagnostic LP in all MS cases, denoted with a *circle*, in relation to **a** Bayesian risk estimate for multiple sclerosis (BREMS), **b** progression index (PI), and **c** multiple sclerosis severity scale (MSSS) scores, after median 7 years of disease duration. The correlation between lactate CSF levels and BREMS however lost its significance after correcting the results for the effect of age and gender

A negative correlation was found between lactate levels and disease duration in MS patients (*r*_s_ = −0.21; *p* = 0.008). In contrast, lactate levels did not correlate with clinical disability assessed by the EDSS at the time of CSF withdrawal. At long-term follow-up, there were significant correlations between lactate levels at diagnosis and BREMS (*r*_s_ = 0.152; *p* = 0.048), PI (*r*_s_ = 0.227; *p* = 0.006), and MSSS (*r*_s_ = 0.178; p = 0.026) scores among all MS cases (Fig. 2).

After entering age and gender as control variables in a partial correlation analysis, CSF lactate levels at the time of LP still showed a significant correlation with PI (*r* = 0.36; *p* < 0.001) and MSSS (*r* = 0.22; *p* = 0.02, one-tailed). Conversely, after correction for age and gender, CSF lactate correlation with BREMS lost its significance.

To better investigate the correlation between lactate and the inflammatory process typical of MS, RRMS subjects were categorized according to the absence (Gd−; *n* = 60) or the presence (Gd+; *n* = 58) of contrast-enhancing lesions at baseline MRI. Our analysis showed that CSF levels of lactate were similar in the Gd+ group compared to the Gd− group (*p* = n.s.), suggesting no involvement of this biomarker in the acute stage of inflammation. In addition, non-significant correlations were found between CSF lactate levels, CSF leukocyte count, or relapse rate. Furthermore, lactate levels were similar among patients categorized according to the presence of oligoclonal bands (present *n* = 87; absent *n* = 31) (not shown).

### CSF lactate levels correlate with neuronal markers of axonal damage

**Fig. 3:**
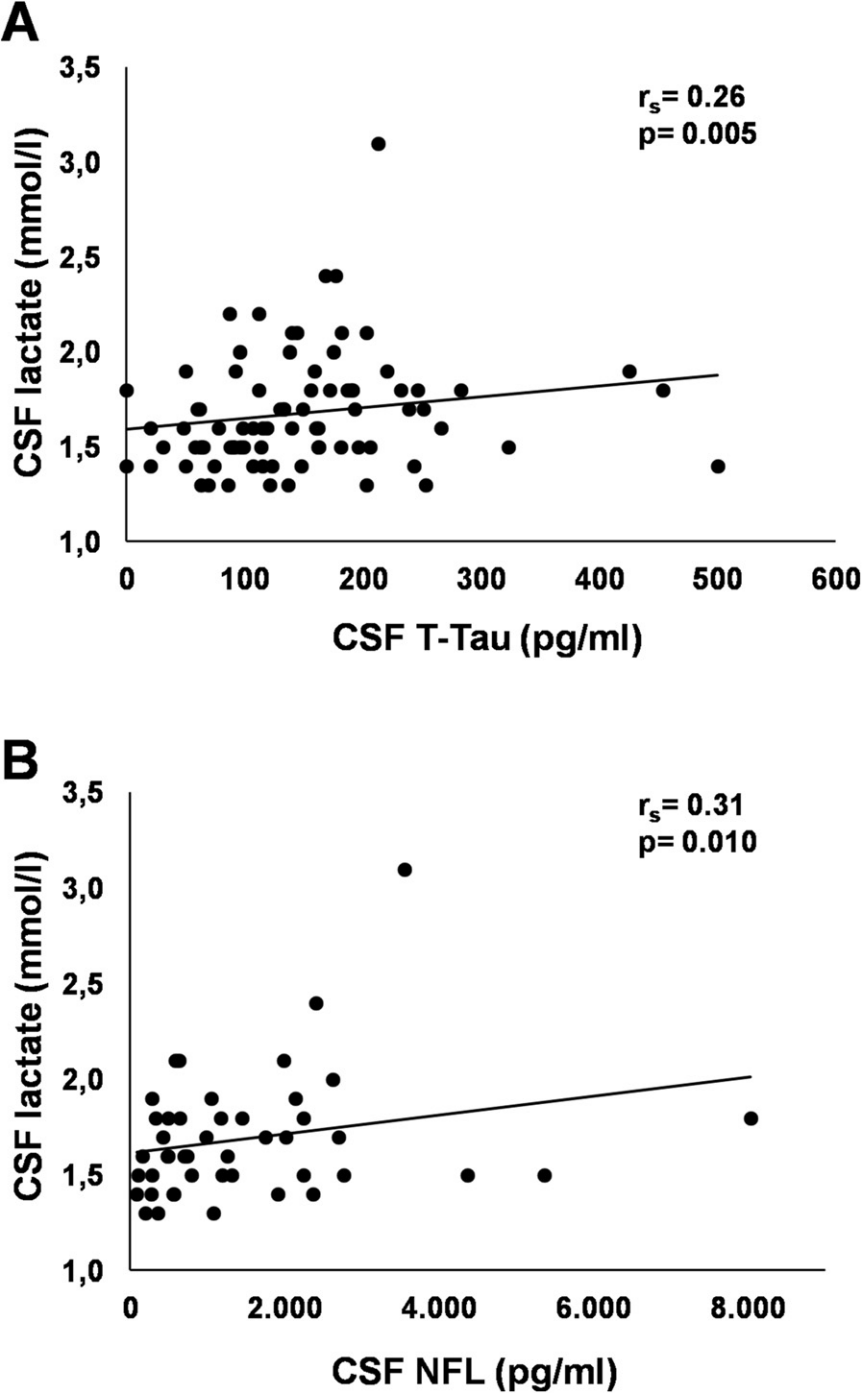
CSF lactate levels correlate with neuronal markers of axonal damage. CSF lactate levels in relation to **a** total tau protein (*t-tau*) and **b** neurofilament light protein (*NFL*), collected during diagnostic LP in all MS cases, denoted with a *circle*. Correlations between lactate and CSF levels of both T-tau and NFL however lost their significance

The CSF levels of lactate showed a positive correlation with t-tau (*r*_s_ = 0.263; *p* = 0.005) and NFL (*r*_s_ = 0.305; *p* = 0.01), well-recognized markers of neuronal damage (Fig. 3). However, after correcting for age and gender, the correlation of CSF lactate with t-tau and NFL lost its significance.

## Discussion

Our study shows a correlation between CNS energy metabolism, as measured by means of CSF lactate concentration, and MS disease severity.

In the last decade, increasing evidence suggests that mitochondrial dysfunction and concomitant oxidative damage play a role in the pathogenesis of MS, leading to an energy imbalance and driving neuroaxonal degeneration [5–7, 19]. Under normal homeostatic conditions, brain lactate is produced through anaerobic glycolysis by neurons to meet acutely increased energy demands and by astrocytes to be shuttled to neurons as a substrate for mitochondrial oxidative metabolism [20, 21]. It is well known that measurement of CSF lactate concentration is a useful biomarker in mitochondrial disorders, since increased CSF lactate levels may represent the result of accumulating energetic metabolites due to mitochondrial dysfunction [22]. Moreover, the concentration of cerebral lactate directly depends on its rate of production in the brain, because blood and CSF lactate concentrations are independent from one another [23, 24].

Indeed, data evaluating the CSF lactate levels in MS patients are not consistent across the studies, most probably due to the small size and high variability of patient cohorts. Proton-magnetic resonance spectroscopy (1H MRS) studies showed that MS patients have higher levels of CSF lactate [12, 13]. MRI studies showed a correlation between CSF lactate concentration and the number of inflammatory plaques [8, 11]. In contrast, data reporting decreased CSF lactate levels in the early stages of MS or comparable concentration have also been published [9, 10, 25]. In our study, carried out in a large cohort of RRMS patients, we noted a significant increase of CSF lactate levels, possibly due to the deranged use of energetic substrates caused by the impairment of oxidative phosphorylation cycle.

No significant correlation was observed between CSF lactate levels and the number of contrast-enhancing lesions, IgG index, CSF leukocyte count, or relapse, suggesting that increased anaerobic pathways and acute inflammation are independent events and supporting the hypothesis that focal inflammation and neurodegeneration may disconnect early in the disease course [26].

Interestingly, CSF lactate correlated significantly with PI, MSSS, and BREMS scores, suggesting an association between altered energy metabolism and MS severity, that may start during the relapsing-remitting phase of the disease. Moreover, we observed that CSF lactate concentrations significantly correlated with age both in patients and controls, indicating an age-related increase in CSF lactate independent of MS pathology [27]. Age at onset is considered a prognostic factor of MS severity; it may thus represent a possible confounding factor limiting the significance of our results. However, after correcting for age and gender, CSF lactate still significantly correlated with clinical scores of disease severity measured up to 5 years later, indicating that energy metabolism in the brain is related to MS severity apart from age. Conversely, after correction for age and gender, CSF lactate correlations with BREMS, t-tau, and NFL lost their significance. This result may indicate that CSF lactate may depend on increased glial and/or neuronal metabolism as expected during MS activity. Indeed, although glucose is usually assumed to be the main energy source for living tissues, there are some indications that lactate is preferentially metabolized by neurons in the brain of several mammalian species [28, 29]. According to the lactate-shuttle hypothesis, glial cells are responsible for transforming glucose into lactate and for providing lactate to the neurons [30, 31].

We found no correlation between CSF lactate levels and neurologic disability as assessed by the EDSS in accordance to previous studies [12].

It is well established that CSF tau and NFL levels represent biomarkers of acute neuroaxonal damage [32]. Experimental studies suggested that tau protein may cause neuronal injury by altering the targeting and function of synaptic mitochondria throughout many mechanisms. In fact, mitochondria are transported along axons by the motor protein kinesin; it seems that tau is able to bind this kinesin and compete with other cargos, preventing their attachment and subsequent transport, that are crucial for proper synaptic activity [33, 34]. Tau may also directly influence mitochondrial function by reducing mitochondrial membrane potential and ATP levels increasing susceptibility to oxidative stress, by affecting the complex I of the respiratory chain or by interfering with both mitochondrial fission and fusion [14, 35].

Remarkably, inflammation and myelin loss in the CNS in MS increase the energy demand of a neuron due to ineffective nerve conductance and thus challenge the mitochondrial machinery [5]. Presumably as a compensatory mechanism, the density of mitochondria as well as the transport velocity in the axons increases in demyelinated neurons [36]. Mitochondria from the motor cortex of MS patients display abnormal reductions in the activity of complexes I and III, which are not limited to the areas of myelin loss [37]. Recently, it has been hypothesized that these mitochondrial changes are amplified in neuronal cell bodies, causing energy failure in axons and driving neurodegeneration early in the disease process [26].

Although not yet proven, it is likely that accumulated neuro-axonal damage may influence per se mitochondrial injury and energy deficiency over time, throughout a potential tau-dependent amplification mechanisms, as reported in other neurodegenerative disorders and tauopathies [14]. The inverse relationship between CSF levels of lactate and disease duration in our MS patients is also consistent with this hypothesis.

Interestingly, NFL reflects acute axonal loss due to inflammatory mechanisms [38]. In fact, levels of NFL were higher in MS patients with relapse activity compared to patients in remission and correlated with the presence of contrast-enhancing lesions or of CSF oligoclonal bands [39, 40]. Increased NFL levels in CSF of RRMS patients were normalized by anti-inflammatory treatment, such as natalizumab [41]. Furthermore, NFL implies prognostic value for conversion from clinically isolated syndrome (CIS) to definite MS [32, 42]. In our study, increased CSF lactate significantly correlated with the concentration of NFL in the CSF of RRMS patients. We proposed that higher CSF levels of NFL in patients with more impaired energy state may reflect subtle and overlapping forms of axonal damage, causing metabolic adaptations that become progressively detrimental for neurons. This hypothesis is also supported by the constant release of NFL into CSF previously found during all stages of MS, even in the absence of relapses or new MRI activity [43].

## Conclusions

Early mitochondrial dysfunction may be reversible and a valuable new target for treatment [44]. The results of the present study point to impaired energy metabolism and mitochondrial function as important mechanisms contributing to disease severity in MS, supporting the concept that therapies aimed at ameliorating mitochondrial injury might be helpful to prevent disability accumulation/progression in MS patients.

## References

[1] Barkhof F, Calabresi PA, Miller DH, Reingold SC (2009). Imaging outcomes for neuroprotection and repair in multiple sclerosis trials. Nat Rev Neurol.

[2] Frischer JM, Bramow S, Dal-Bianco A, Lucchinetti CF, Rauschka H, Schmidbauer M (2009). The relation between inflammation and neurodegeneration in multiple sclerosis brains. Brain.

[3] Filippi M, Bozzali M, Rovaris M, Gonen O, Kesavadas C, Ghezzi A (2003). Evidence for widespread axonal damage at the earliest clinical stage of multiple sclerosis. Brain.

[4] Lassmann H, Brück W, Lucchinetti CF (2007). The immunopathology of multiple sclerosis: an overview. Brain Pathol.

[5] Trapp BD, Stys PK (2009). Virtual hypoxia and chronic necrosis of demyelinated axons in multiple sclerosis. Lancet Neurol.

[6] Campbell GR, Mahad DJ (2011). Mitochondria as crucial players in demyelinated axons: lessons from neuropathology and experimental demyelination. Autoimmune Dis.

[7] Su K, Bourdette D, Forte M (2013). Mitochondrial dysfunction and neurodegeneration in multiple sclerosis. Front Physiol.

[8] Simone IL, Federico F, Trojano M, Tortorella C, Liguori M, Giannini P (1996). High resolution proton MR spectroscopy of cerebrospinal fluid in MS patients. Comparison with biochemical changes in demyelinating plaques. J Neurol Sci.

[9] Aasly J, Gårseth M, Sonnewald U, Zwart JA, White LR, Unsgård G (1997). Cerebrospinal fluid lactate and glutamine are reduced in multiple sclerosis. Acta NeurolScand.

[10] Schocke MF, Berger T, Felber SR, Wolf C, Deisenhammer F, Kremser C (2003). Serial contrast-enhanced magnetic resonance imaging and spectroscopic imaging of acute multiple sclerosis lesions under high-dose methylprednisolone therapy. Neuroimage.

[11] Lutz NW, Viola A, Malikova I, Confort-Gouny S, Audoin B, Ranjeva JP (2007). Inflammatory multiple-sclerosis plaques generate characteristic metabolic profiles in cerebrospinal fluid. PLoS One.

[12] Regenold WT, Phatak P, Makley MJ, Stone RD, Kling MA (2008). Cerebrospinal fluid evidence of increased extra-mitochondrial glucose metabolism implicates mitochondrial dysfunction in multiple sclerosis disease progression. J NeurolSci.

[13] Zaaraoui W, Rico A, Audoin B, Reuter F, Malikova I, Soulier E (2010). Unfolding the long-term pathophysiological processes following an acute inflammatory demyelinating lesion of multiple sclerosis. Magn Reson Imaging.

[14] Schulz KL, Eckert A, Rhein V, Mai S, Haase W, Reichert AS (2012). A new link to mitochondrial impairment in tauopathies. Mol Neurobiol.

[15] Polman CH, Reingold SC, Edan G, Filippi M, Hartung HP, Kappos L (2005). Diagnostic criteria for multiple sclerosis: 2005 revisions to the “McDonald criteria”. Ann Neurol.

[16] Kurtzke JF (1983). Rating neurologic impairment in multiple sclerosis: an expanded disability status scale (EDSS). Neurology.

[17] Roxburgh RH, Seaman SR, Masterman T, Hensiek AE, Sawcer SJ, Vukusic S (2005). Multiple sclerosis severity score: using disability and disease duration to rate disease severity. Neurology.

[18] Bergamaschi R, Quaglini S, Trojano M, Amato MP, Tavazzi E, Paolicelli D (2007). Early prediction of the long term evolution of multiple sclerosis: the Bayesian risk estimate for multiple sclerosis (BREMS) score. J Neurol Neurosurg Psychiatry.

[19] Stadelmann C (2011). Multiple sclerosis as a neurodegenerative disease: pathology, mechanisms and therapeutic implications. Curr Opin Neurol.

[20] Fünfschilling U, Supplie LM, Mahad D, Boretius S, Saab AS, Edgar J (2012). Glycolytic oligodendrocytes maintain myelin and long-term axonal integrity. Nature.

[21] Karus C, Ziemens D, Rose CR (2015). Lactate rescues neuronal sodium homeostasis during impaired energy metabolism. Channels (Austin).

[22] Finsterer J (2001). Cerebrospinal-fluid lactate in adult mitochondriopathy with and without encephalopathy. Acta Med Austriaca.

[23] Fishman RA (1993). Cerebrospinal fluid in diseases of the nervous system.

[24] Sommer JB, Gaul C, Heckmann J, Neundörfer B, Erbguth FJ (2002). Does lumbar cerebrospinal fluid reflect ventricular cerebrospinal fluid? A prospective study in patients with external ventricular drainage. Eur Neurol.

[25] FonalledasPerelló MA, Politi JV, Dallo Lizarraga MA, Cardona RS (2008). The cerebrospinal fluid lactate is decreased in early stages of multiple sclerosis. P R Health Sci J.

[26] Friese MA, Schattling B, Fugger L (2014). Mechanisms of neurodegeneration and axonal dysfunction in multiple sclerosis. Nat Rev Neurol.

[27] Currais A (2015). Ageing and inflammation—a central role for mitochondria in brain health and disease. Ageing Res Rev.

[28] Zilberter Y, Zilberter T, Bregestovski P (2010). Neuronal activity in vitro and the in vivo reality: the role of energy homeostasis. Trends Pharmacol Sci.

[29] Wyss MT, Jolivet R, Buck A, Magistretti PJ, Weber B (2011). In vivo evidence for lactate as a neuronal energy source. J Neurosci.

[30] Gladden LB (2004). Lactate metabolism: a new paradigm for the third millennium. J Physiol Lond.

[31] Pellerin L, Bouzier-Sore AK, Aubert A (2007). Activity-dependent regulation of energy metabolism by astrocytes: an update. Glia.

[32] Martínez MA, Olsson B, Bau L, Matas E, CoboCalvo Á, Andreasson U (2015). Glial and neuronal markers in cerebrospinal fluid predict progression in multiple sclerosis. Mult Scler.

[33] Utton MA, Noble WJ, Hill JE, Anderton BH, Hanger DP (2005). Molecular motors implicated in the axonal transport of tau and alpha-synuclein. J Cell Sci.

[34] Cuchillo-Ibanez I, Seereeram A, Byers HL, Leung KY, Ward MA, Anderton BH (2008). Phosphorylation of tau regulates its axonal transport by controlling its binding to kinesin. FASEB J.

[35] DuBoff B, Götz J, Feany MB (2012). Tau promotes neurodegeneration via DRP1 mislocalization in vivo. Neuron.

[36] Kiryu-Seo S, Ohno N, Kidd GJ, Komuro H, Trapp BD (2010). Demyelination increases axonal stationary mitochondrial size and the speed of axonal mitochondrial transport. J Neurosci.

[37] Dutta R, McDonough J, Yin X, Peterson J, Chang A, Torres T (2006). Mitochondrial dysfunction as a cause of axonal degeneration in multiple sclerosis patients. Ann Neurol.

[38] Villar LM, Picón C, Costa-Frossard L, Alenda R, García-Caldentey J, Espiño M (2015). Cerebrospinal fluid immunological biomarkers associated with axonal damage in multiple sclerosis. Eur J Neurol.

[39] Norgren N, Sundström P, Svenningsson A, Rosengren L, Stigbrand T, Gunnarsson M (2004). Neurofilament and glial fibrillary acidic protein in multiple sclerosis. Neurology.

[40] Teunissen CE, Khalil M (2012). Neurofilaments as biomarkers in multiple sclerosis. Mult Scler.

[41] Gunnarsson M, Malmeström C, Axelsson M, Dahle C, Vrethem M, Olsson T (2011). Axonal damage in relapsing multiple sclerosis is markedly reduced by natalizumab. Ann Neurol.

[42] Teunissen CE, Iacobaeus E, Khademi M, Brundin L, Norgren N, Koel-Simmelink MJ (2009). Combination of CSF N-acetylaspartate and neurofilaments in multiple sclerosis. Neurology.

[43] Malmeström C, Haghighi S, Rosengren L, Andersen O, Lycke J (2003). Neurofilament light protein and glial fibrillary acidic protein as biological markers in MS. Neurology.

[44] Vawter MP, Tomita H, Meng F, Bolstad B, Li J, Evans S (2006). Mitochondrial-related gene expression changes are sensitive to agonal-pH state: implications for brain disorders. Mol Psychiatry.

